# Association of hepatic steatosis and liver fibrosis with chronic obstructive pulmonary disease among adults

**DOI:** 10.1038/s41598-024-61696-x

**Published:** 2024-05-11

**Authors:** Dayang Zheng, Xiang Liu, Wei Zeng, Wangyan Zhou, Chunxiang Zhou

**Affiliations:** 1 Department of Thoracic Surgery, East Hospital, The Second Affiliated Hospital, Hengyang Medical School, University of South China, No. 30 Jiefang Road, Shigu District, Hengyang, 421009 Hunan Province China; 2 Department of Medical Record, The First Affiliated Hospital, Hengyang Medical School, University of South China, Hengyang, 421001 China

**Keywords:** COPD, Hepatic steatosis, NAFLD, NHANES, VCTE, Endocrinology, Endocrine system and metabolic diseases, Growth disorders, Metabolic syndrome, Endocrine system and metabolic diseases, Metabolic disorders, Respiratory tract diseases, Chronic obstructive pulmonary disease

## Abstract

With high prevalence and substantial mortality, metabolic dysfunction-associated steatotic liver disease and chronic obstructive pulmonary disease (COPD) are significant public health concerns. Utilizing a large, population-based dataset from the National Health and Nutrition Examination Survey, our study probes the relationship between COPD prevalence and hepatic steatosis and fibrosis, as measured by Vibration-Controlled Transient Elastography. We analyzed data from 693 individuals with COPD and 7229 without. Through weighted multivariate logistic regression analysis, a restricted cubic spline curve, and threshold effect analysis, we investigated the correlation between the severity of hepatic steatosis and fibrosis and the presence of COPD. Our findings revealed a positive correlation between the controlled attenuation parameter (CAP) and COPD prevalence [OR = 1.03 (95% CI 1.01, 1.05)], even after multivariate adjustment. Furthermore, we observed a U-shaped association between CAP and COPD, where the inflection point, CAP value of 264.85 dB/m, corresponded to the lowest COPD prevalence. Our study emphasizes a substantial and complex link between hepatic steatosis and COPD. These findings urge healthcare professionals to factor liver health into COPD management and prompt further exploration into the underlying mechanisms. This could pave the way for the development of improved prevention and treatment strategies.

## Introduction

Chronic Obstructive Pulmonary Disease (COPD) poses a significant challenge to global public health, characterized by persistent respiratory symptoms and airflow limitation that lead to chronic morbidity and mortality across the globe^1–4^. This condition is frequently complicated by various comorbidities, thereby increasing its severity and the risk of fatal outcomes^5,6^. Among these comorbidities, Metabolic Dysfunction-Associated Steatotic Liver Disease (MASLD), formerly recognized as Non-Alcoholic Fatty Liver Disease (NAFLD), is rapidly emerging as a global health concern, now affecting up to 30% of the global population, with variations across different regions^7,8^. A recent study from the United States has shown that the prevalence of MASLD has exceeded 32%^9^. A pivotal study by Tsutsumi et al. illustrates that respiratory function, including the prevalence of COPD, is comparably affected in MASLD and NAFLD patients, underscoring the significant overlap in these conditions^10^. This highlights the necessity of addressing MASLD alongside COPD in global health strategies.

MASLD is defined by the excessive accumulation of hepatic triglycerides, absent any significant alcohol intake or secondary etiologies^11,12^. This liver condition exhibits a multifaceted bidirectional relationship with metabolic syndrome, positioning it as an independent prognosticator for escalating liver-specific and overall mortality rates^13^. Recent investigations have even postulated MASLD as a novel independent risk factor for cardiovascular disease^14,15^.

Pertinently, COPD patients often demonstrate an augmented visceral fat profile, which is linked to systemic inflammatory processes and cardiometabolic co-morbidities^16^. This may be attributed to pulmonary dysfunction and dyspnea, which limit physical activity, promote a sedentary lifestyle, and consequently result in the accumulation of visceral adipose tissue^17,18^. However, the scientific literature remains deficient in comprehensive studies exploring the connection between COPD and the extent of hepatic steatosis and fibrosis^19^. While initial studies have begun to probe into this association, they remain encumbered by limitations such as small sample sizes and diagnostic inaccuracies due to reliance on blood composite indices for assessing hepatic steatosis and fibrosis^20^.

Vibration-Controlled Transient Elastography (VCTE) has emerged as a validated, non-invasive technique with high accuracy for assessing hepatic steatosis and fibrosis^21,22^. This research endeavor aims to explore the relationship between the degree of hepatic steatosis and fibrosis, as gauged by VCTE, with the prevalence of COPD. This investigation will leverage a substantial population-based dataset from the National Health and Nutrition Examination Survey (NHANES). It is our aspiration that this study will unveil novel research perspectives and strategies, thereby augmenting the prevention and treatment paradigms for COPD.

## Methods

### Data source

**Figure 1:**
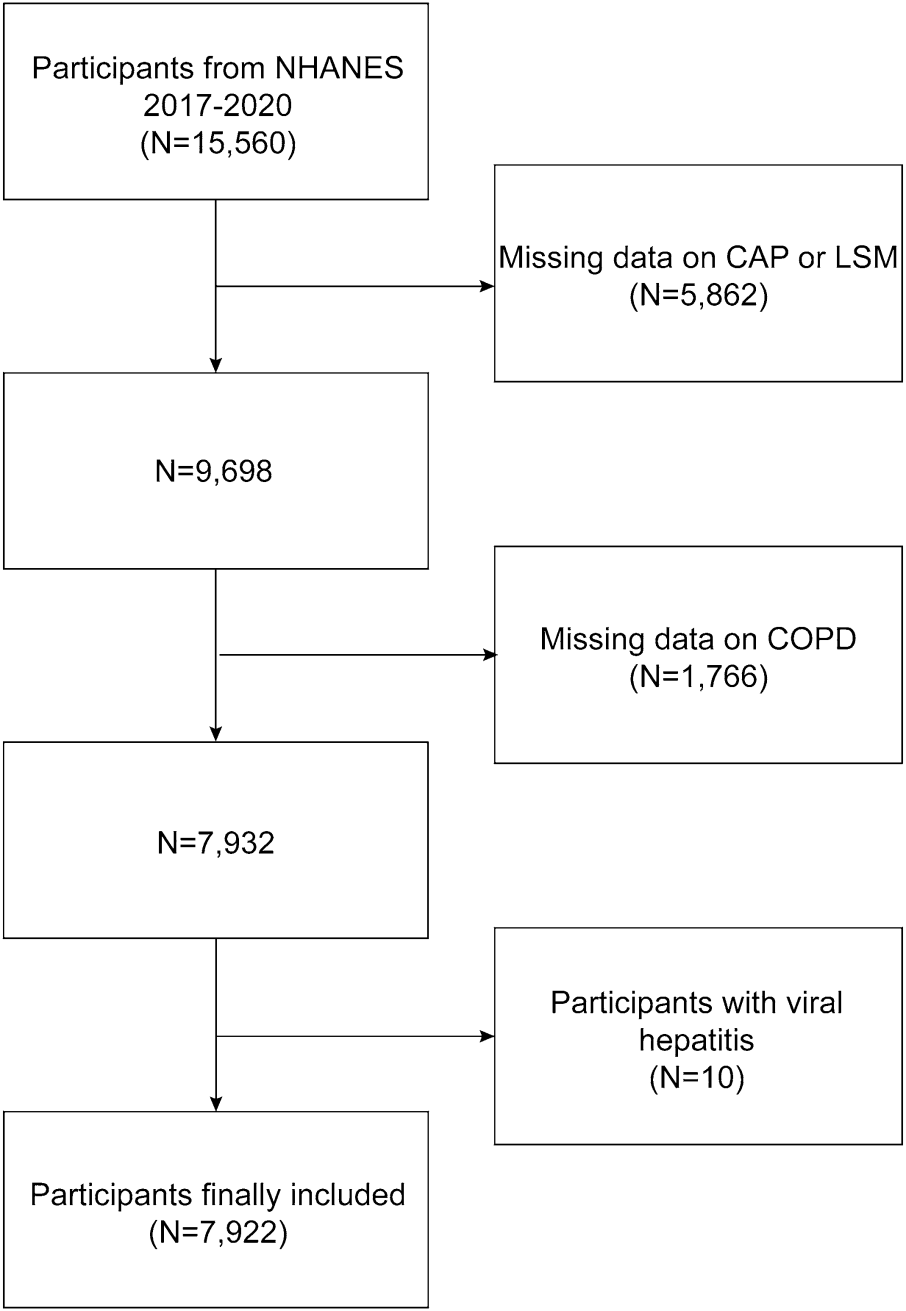
Flow chart of participant’s selection. NHANES, National Health and Nutrition Examination Survey.

The National Center for Health Statistics (NCHS) conducted the National Health and Nutrition Examination Survey (NHANES), a nationally representative survey of the non-institutionalized population in the United States, as the basis for this study^23,24^. All study procedures were approved by the Research Ethics Review Board of NCHS and informed consent has been obtained from all participants. The dataset used in this study includes data from January 2017 to March 2020. In this study, after excluding participants with missing Controlled Attenuation Parameter (CAP) or Liver Stiffness Measurement (LSM) (n = 5862), missing COPD data (n = 1766), and baseline viral hepatitis (n = 10), a total of 7922 samples were enrolled for the final analysis (Fig. 1).

### CAP and LSM

For the assessment of hepatic steatosis and fibrosis, we utilized Vibration-Controlled Transient Elastography (VCTE) with the FibroScan device from EchoSens, France. This non-invasive technology allowed for the precise measurement of CAP and LSM. Each participant underwent a minimum of ten measurements conducted by an experienced operator. The median CAP value was automatically calculated by the device. Additionally, LSM was determined using the device's integrated software, which is based on established protocols for evaluating the degree of fibrosis^25,26^.

### COPD

Participants were classified as having COPD if they reported ever being told by a healthcare professional that they had COPD, chronic bronchitis, or emphysema. This classification method has been widely applied in previous NHANES-based research for identifying individuals with COPD.

### Covariates

This study incorporated several covariates, including age, gender, and race. Socioeconomic status was represented by the ratio of income to poverty (PIR). Biological variables included aspartate transferase, BMI (body mass index), alkaline phosphatase, and aspartate aminotransferase. Lifestyle factors such as vigorous activities, smoking status, and alcohol drinking status were also taken into account. Additionally, disease states like diabetes, high blood pressure, and asthma status were included as covariates.

### Statistical analysis

All variables in this study had missing values between 0 and 8.6%, with the most missing variable being vigorous activities (8.6%). Missing variables were interpolated by the R package 'missForest' (version: 1.6), which is based on the random forest approach. During the descriptive analysis phase, we represented continuous variables using mean values and standard deviations (SD), whereas categorical variables were expressed through frequencies and percentages. The group differences between the COPD and non-COPD groups were analyzed using the Chi-square test for categorical data and the T-test for continuous data. For examining the linear relationship between COPD with CAP and LSM, we used weighted multivariate logistic regression analysis. Additionally, subgroup analysis was carried out to investigate the relationship between COPD with CAP and LSM in various subpopulations based on factors such as sex, race, education, BMI, and diabetes status. In addition, multinomial logistic regression model was used to investigate the association between CAP and GOLD-graded COPD. To investigate the nonlinear association between COPD with CAP and LSM, we used restricted cubic spline (RCS) curve^27,28^. We established statistical significance at two-sided *P* < 0.05^22,24^. The statistical software packages used were R (version 4.2) and Python (version 3.10.4).

### Ethical approval and consent to participate

The portions of this study involving human participants, human materials, or human data were conducted in accordance with the Declaration of Helsinki and were approved by the NCHS Ethics Review Board. The patients/participants provided their written informed consent to participate in this study.

## Results

### Baseline characteristics

**Figure 2:**
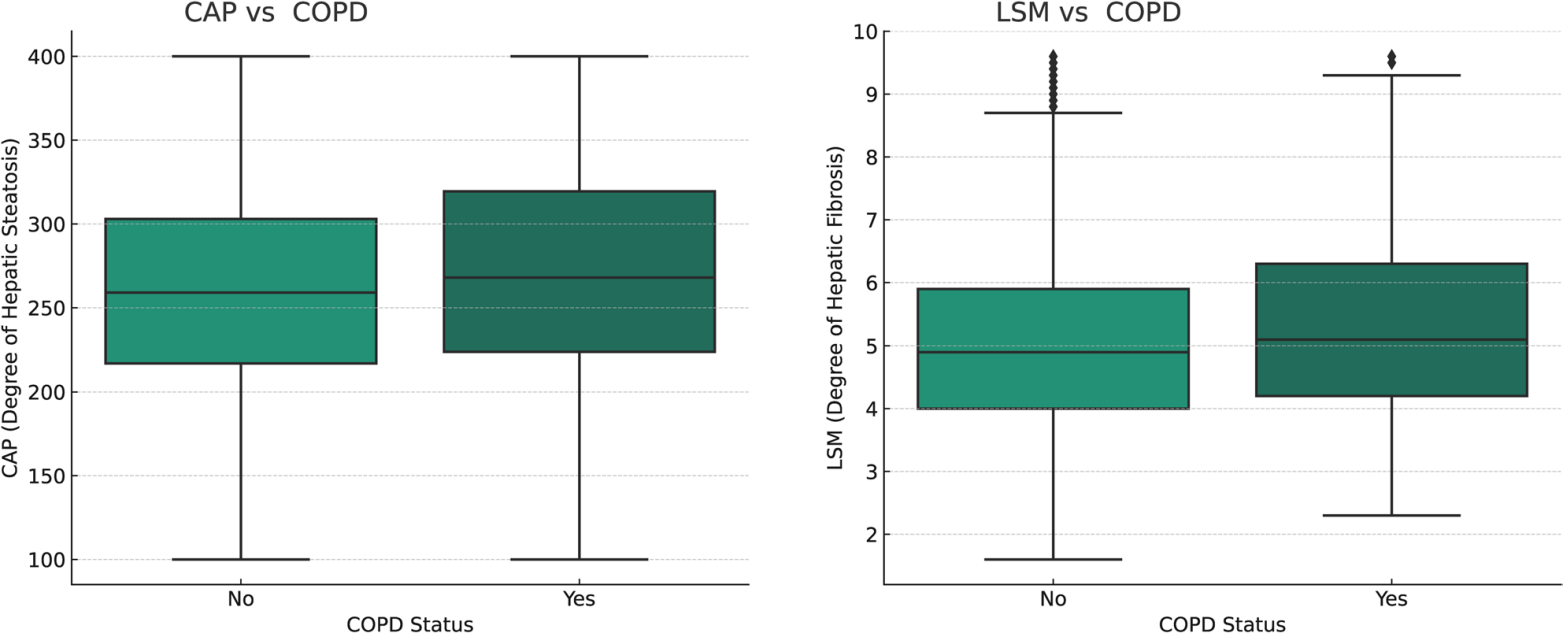
Relationships between CAP, LSM, and COPD. Left panel: Box plot showing the distribution of CAP (degree of hepatic steatosis) in patients with COPD status 'No' and 'Yes'. The central line in the box represents the median. The top and bottom of the box represent the upper and lower quartiles, respectively. The whiskers extend to the most extreme data points not considered outliers. Right panel: Box plot showing the distribution of LSM (degree of hepatic fibrosis) in patients with COPD status 'No' and 'Yes'. The central line in the box represents the median. The top and bottom of the box represent the upper and lower quartiles, respectively. The whiskers extend to the most extreme data points not considered outliers.

In this study, the analysis dataset consisted of data from 693 COPD patients and 7229 non-COPD individuals. The mean age of COPD patients was 60.13 ± 15.24 years, and 47.47% of them were male. The average value of CAP is 277.43 ± 67.27 dB/m, and the average value of LSM is 6.58 ± 5.09 kPa. The baseline characteristics of the study population are presented by presence or absence of COPD and are detailed in Table 1 and Fig. 2.

**Table 1:** Basic characteristics of participants by COPD among U.S. adults.

Characteristics	Total (7922)	COPD (693)	Non-COPD (7229)	*P* value
Age (years)	50.74 ± 17.31	60.13 ± 15.24	49.84 ± 17.23	< 0.001
Sex, n (%)				< 0.001
Male	3914 (49.39)	329 (47.47)	2584 (49.58)	
Female	4009 (50.61)	364 (52.53)	3645 (50.42)	
Race/ethnicity, n (%)				< 0.001
Non-Hispanic White	2707 (34.17)	372 (53.68)	2335 (32.30)	
Non-Hispanic Black	2109 (26.62)	157 (22.66)	1952 (27.00)	
Mexican American	937 (11.83)	31 (4.47)	906 (12.53)	
Other race/multiracial	2169 (27.38)	133 (19.19)	2036 (28.16)	
Education level, n (%)				< 0.001
Less than high school	1469 (18.54)	157 (22.66)	1312 (18.15)	
High school	1918 (24.21)	217 (31.31)	1701 (23.53)	
More than high school	4535 (57,15)	319 (46.03)	4216 (58.32)	
Asthma, n (%)				< 0.001
Yes	1240 (15.65)	296 (42.71)	1857 (13.06)	
No	6682 (84.35)	397 (57.29)	5372 (86.94)	
Diabetes, n (%)				< 0.001
Yes	1412 (17.82)	215 (31.02)	1197 (16.56)	
No	6510 (82.18)	478 (68.98)	6032 (83.44)	
High blood pressure, n (%)				< 0.001
Yes	3024 (38.17)	398 (57.43)	2626 (36.33)	
No	4898 (61.83)	295 (42.57)	4603 (63.67)	
Vigorous activities, n (%)				0.508
Yes	2043 (25.79)	186 (26.84)	1857 (25.69)	
No	5879 (74.21)	507 (73.16)	5372 (74.31)	
Smoking status, (%)				< 0.001
Current smoker	1526 (19.26)	226 (32.61)	1300 (17.98)	
Former smoker	2369 (29.90)	281 (40.55)	2088 (28.88)	
Never smoked	4027 (50.82)	186 (26.84)	3841 (53.13)	
Drinking alcohol, n (%)				< 0.001
Never	682 (8.61)	32 (4.86)	650 (9.51)	
Ever	6813 (81.39)	451 (95.14)	5296 (90.49)	
PIR	2.61 ± 1.63	2.04 ± 1.43	2.67 ± 1.63	< 0.001
BMI (kg/m^2^)	29.93 ± 7.42	31.34 ± 9.03	29.80 ± 7.23	< 0.001
ALT (U/L)	22.35 ± 18.68	19.94 ± 13.47	22.58 ± 19.09	< 0.001
AST (U/L)	21.92 ± 12.36	21.07 ± 12.25	22.00 ± 14.55	< 0.001
ALP (U/L)	77.87 ± 25.94	84.36 ± 28.08	77.25 ± 25.64	< 0.001
CAP (dB/m)	265.53 ± 62.79	277.43 ± 67.27	264.39 ± 62.23	< 0.001
LSM (kPa)	6.04 ± 5.18	6.58 ± 5.09	5.99 ± 5.18	< 0.001

Mean ± SD for continuous variables: the P value was calculated by the weighted linear regression model.

(%) for categorical variables: the P value was calculated by the weighted chi-square test.

*PIR* the ratio of income to poverty, *BMI* body mass index, *COPD* chronic obstructive pulmonary disease, *ALT* alanine transaminase, *AST* aspartate transferase, *ALP* alkaline phosphatase, *CAP* controlled Attenuation Parameter, *LSM* liver stiffness measure.

Based on COPD status comparisons, COPD patients were more likely to be non-Hispanic white, have hypertension, have diabetes, have asthma, ever smoke and drink alcohol, and have higher levels of hepatic steatosis (CAP) and liver fibrosis (LSM) (all *P* < 0.001). The socioeconomic status of COPD patients was generally low, with 22.66% of them receiving less than high school education, and the mean PIR was only 2.04 ± 1.43 (2.67 ± 1.63 for non-COPD participants) (all *P* < 0.001).

### Association between COPD with CAP and LSM

The outcomes of the multivariate logistic regression model, examining the relationship between COPD and the extent of hepatic steatosis as well as liver fibrosis, are displayed in Table 2 and Supplemental Table S1. Without adjusting for any covariates, each 10 dB/m increase in CAP was associated with a 3% increase in the prevalence of COPD [OR = 1.03 (95% CI 1.02, 1.05)], and each 10 kPa increase in LSM was associated with a 3% increase in the prevalence of COPD was associated with an 18% increase in rate [OR = 1.18 (95% CI 1.05, 1.32)].

**Table 2:** The associations between COPD with CAP and LSM.

Exposure	Model 1 [OR (95% CI)]	Model 2 [OR (95% CI)]	Model 3 [OR (95% CI)]
CAP (per 10 dB/m)	1.03 (1.02, 1.05)	1.04 (1.03, 1.06)	1.03 (1.01, 1.05)
CAP (quartile)			
Quartile 1	Reference	Reference	Reference
Quartile 2	1.13 (0.89, 1.42)	1.05 (0.83, 1.32)	0.98 (0.79, 1.27)
Quartile 3	1.09 (0.86, 1.38)	1.11 (0.88, 1.44)	0.85 (0.64, 1.09)
Quartile 4	1.68 (1.35, 2.08)	1.55 (1.22, 1.88)	1.35 (1.12, 1.58)
P for trend	< 0.001	< 0.001	0.031
LSM (per 10 kPa)	1.18 (1.05, 1.32)	1.15 (1.02, 1.28)	1.10 (0.97, 1.22)
LSM (quartile)			
Quartile 1	Reference	Reference	Reference
Quartile 2	1.16 (0.92, 1.48)	1.18 (0.94, 1.49)	1.11 (0.85, 1.43)
Quartile 3	1.21 (0.95, 1.54)	1.22 (0.96, 1.55)	1.10 (0.86, 1.41)
Quartile 4	1.77 (1.41, 2.22)	1.80 (1.46, 2.22)	1.24 (0.96, 1.61)
P for trend	< 0.001	< 0.001	0.046

Model 1: no covariates were adjusted. Model 2: age, gender, race, and smoking status were adjusted. Model 3: age, gender, race, BMI, smoking status, ALT, ALP, AST, diabetes, PIR, high blood pressure, asthma, vigorous activities, and alcohol drinking were adjusted.

*PIR* the ratio of income to poverty, *BMI* body mass index, *COPD* chronic obstructive pulmonary disease, *ALT* alanine transaminase, *AST* aspartate transferase, *ALP* alkaline phosphatase, *CAP* controlled Attenuation Parameter, *LSM* liver stiffness measure.

However, the relationship between CAP and COPD was attenuated but still significant in both partially and fully adjusted models. In the fully adjusted model, with each 10 dB/m increase associated with a 3% increase in the prevalence of COPD [OR = 1.03 (95% CI 1.01, 1.05)]. The relationship between LSM and COPD became insignificant [OR = 1.10 (95% CI 0.97, 1.22)] in partially adjusted and fully adjusted models.

Both CAP and LSM were classified in quartiles. For CAP, the fourth quartile showed a significantly increased risk of COPD in all models compared to the first quartile (reference group), especially in the fully adjusted model with a 35% increase in the prevalence of COPD [OR = 1.35 (95% CI 1.12, 1.58)]. For LSM, compared with the first quartile (reference group), the fourth quartile showed a significantly increased prevalence of COPD in models without adjustment for all covariates and in partially adjusted models, but not in fully adjusted model [OR = 1.24 (95% CI 0.96, 1.61)].

In subgroup analyzes according to sex, race, age, smoking status, education level, BMI, and diabetes status, the associations of CAP and LSM with COPD were consistent across multiple subgroups (*P* for interaction > 0.05) (Table 3). In particular, the increase in CAP associated with an increase in the prevalence of COPD was more pronounced in men, those over 60 years of age, current smoker, non-Hispanic blacks and other races, those with less than high school education, those with a BMI of 25–29.9 kg/m^2^, and those with diabetes.

**Table 3:** Subgroup analysis of the association between COPD with CAP and LSM.

Subgroup	CAP (per 10 dB/m) [OR (95%CI)]	P for interaction	LSM (per 10 kPa) [OR (95%CI)]	*P* for interaction
Gender		0.193		0.622
Male	1.03 (1.01, 1.05)		1.02 (0.88, 1.16)	
Female	1.02 (1.00, 1.04)		0.96 (0.83, 1.07)	
Age		0.215		0.247
< 60 years	1.03 (1.00, 1.06)		0.96 (0.87, 1.05)	
≥ 60 years	1.04 (1.01, 1.07)		1.02 (0.92, 1.11)	
Race/ethnicity		0.433		0.155
Non-Hispanic White	1.03 (1.01, 1.05)		1.02 (0.95, 1.11)	
Non-Hispanic Black	1.04 (1.02, 1.06)		1.04 (0.91, 1.17)	
Mexican American	1.02 (0.98, 1.06)		0.97 (0.87, 1.09)	
Other race	1.03 (1.02, 1.05)		0.96 (0.85, 1.07)	
Smoking status		0.380		0.191
Current smoker	1.04 (1.01, 1.07)		1.03 (0.92, 1.14)	
Former smoker	1.02 (1.00, 1.04)		1.01 (0.92, 1.10)	
Never smoked	1.02 (1.01, 1.03)		0.99 (0.95, 1.03)	
Education level		0.177		0.513
Less than high school	1.05 (1.00, 1.09)		0.89 (0.83, 0.96)	
High school	1.02 (0.99, 1.06)		0.95 (0.89, 1.02)	
More than high school	1.03 (1.00, 1.06)		1.01 (0.93, 1.10)	
BMI		0.115		0.112
< 24.9 kg/m^2^	1.01 (0.95, 1.07)		1.02 (0.95, 1.09)	
25–29.9 kg/m^2^	1.03 (1.01, 1.05)		1.00 (0.91, 1.08)	
≥ 30 kg/m^2^	1.05 (0.98, 1.14)		0.98 (0.89, 1.09)	
Diabetes		0.166		0.406
No	1.03 (1.00, 1.07)		0.98 (0.88, 1.11)	
Yes	1.05 (1.00, 1.12)		1.01 (0.96, 1.14)	

Age, gender, race, BMI, smoking status, ALT, ALP, AST, diabetes, PIR, high blood pressure, asthma, vigorous activities, and alcohol drinking were adjusted.

*PIR* the ratio of income to poverty, *BMI* body mass index, *COPD* chronic obstructive pulmonary disease, *ALT* alanine transaminase, *AST* aspartate transferase, *ALP* alkaline phosphatase, *CAP* controlled Attenuation Parameter, *LSM* liver stiffness measure.

**Figure 3:**
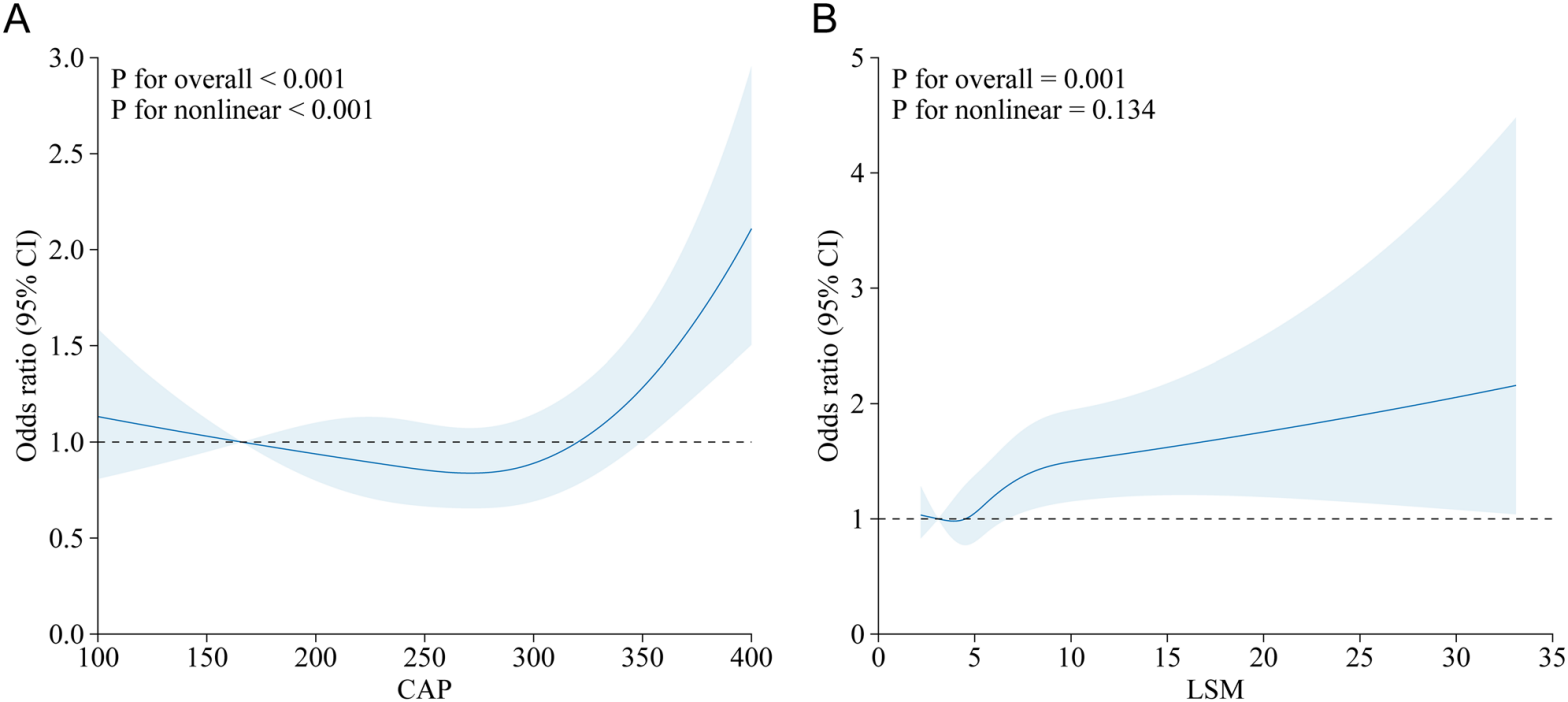
The nonlinear associations between COPD with degree of hepatic steatosis and fibrosis. The solid red line represents the smooth curve fit between variables. Blue bands represent the 95% of confidence interval from the fit. (**A**) CAP and COPD; (**B**) LSM and COPD.

Figure 3 also demonstrates the nonlinear relationship between COPD with CAP and LSM. The outcomes indicate a U-shaped relationship between CAP and COPD. Subsequently, we further investigated the inflection point within the U-shape and the linear associations at both ends using threshold effect analysis and two-segmented linear regression models. The results showed that when CAP was at the inflection point of the U-shaped relationship (264.85 dB/m), the prevalence of COPD was the lowest. When CAP was less than 264.85 dB/m, there was a negative correlation between the degree of liver steatosis and COPD [OR = 1.00 (95% CI 0.99, 1.01)]. However, when CAP was greater than 264.85 dB/m, there was a positive correlation between the degree of liver steatosis and COPD [OR = 1.03 (95% CI 1.01, 1.05)] (Table 4).

**Table4:** Threshold effect analysis of CAP on COPD using the two-piecewise linear regression model.

	Adjusted OR (95% CI)
CAP	
Fitting by the standard linear model	1.03 (1.01, 1.05)
Fitting by the two-piecewise linear model	
Inflection point	264.85
CAP < 272.39 (dB/m)	1.00 (0.99, 1.01)
CAP > 272.39 (dB/m)	1.03 (1.01, 1.05)
Log likelihood ratio	< 0.001

Age, gender, race, BMI, smoking status, ALT, ALP, AST, diabetes, PIR, high blood pressure, asthma, vigorous activities, and alcohol drinking were adjusted.

*COPD* chronic obstructive pulmonary disease, *CAP* controlled Attenuation Parameter.

## Discussion

Our population-based study explored the association between hepatic steatosis and fibrosis, assessed by VCTE, with the prevalence of COPD. We observed a positive association between the degree of hepatic steatosis and the prevalence of COPD, even after multivariate adjustment. Interestingly, our analysis suggested a U-shaped association between CAP and COPD, with the lowest prevalence of COPD at a CAP value of 264.85 dB/m. Below this inflection point, there was a negative correlation between hepatic steatosis and COPD, whereas above this threshold, a positive correlation emerged. This nuanced relationship warrants further exploration to understand the underlying mechanisms and potential implications for COPD management.

Our investigation builds upon and extends the existing literature. A previous cross-sectional analysis by Miao et al. studied the relationship between lung function parameters and fibrosis severity in metabolic associated fatty liver disease (MAFLD) patients and found an independent association between MAFLD and impaired pulmonary function, aligning with our findings^29^. Tsutsumi et al. also found MAFLD to be an independent factor for COPD, suggesting a link via low-grade inflammation^30^. Another study investigating non-alcoholic fatty liver disease (NAFLD) prevalence and severity in patients with COPD showed a substantial prevalence of steatosis, NASH, and fibrosis, and identified obesity and insulin resistance as key contributing factors^18^. Our study provides further support for these associations in a larger population-based sample. The findings of our study are also in line with a recent investigation that assessed the prevalence of NAFLD in a group of 48 patients with COPD^31^. In this study, non-invasive biomarkers and imaging methods were used to determine the prevalence of NAFLD. The detection of steatosis was accomplished by employing magnetic resonance mDIXON-Quant sequence imaging. In contrast, the identification of fibrosis utilized both the acoustic radiation force impulse and the FIB-4 index. It was quite noteworthy that 58.3% of the subjects under examination had a 5% level of fat, with nearly one-fourth of them exceeding 10%. Moreover, the acoustic radiation force impulse indicated that 45.8% of the studied patients possessed severe hepatic fibrosis. The FIB-4 index disclosed that advanced fibrosis was present in 18.75% of the individuals. Despite the absence of a significant statistical relationship between COPD groups and the presence of steatosis and fibrosis (≥ F2) as determined by the acoustic radiation force impulse, a significant link was discovered between the presence of fibrosis and COPD groups when employing the FIB-4 index. Furthermore, it was found that in the groups with more severe COPD, there were elevated levels of γ-glutamyl transferase and alkaline phosphatase, enzymes typically heightened in liver-affecting conditions. The results of this study echo our findings and highlight the significant prevalence of NAFLD in patients with COPD. In addressing the association between COPD and liver conditions such as steatosis and fibrosis, our study underscores the potential benefits of this revelation. Identifying these links offers a pivotal opportunity for early intervention and targeted management strategies, which are crucial in mitigating the progression and impact of COPD. By recognizing liver steatosis and fibrosis as significant comorbidities, healthcare providers can adopt a more holistic approach to patient care, incorporating liver health assessments into regular COPD management protocols. This integrative strategy not only aims to improve patient outcomes but also highlights the need for interdisciplinary collaboration in the treatment of COPD, fostering a comprehensive approach to tackling its multifaceted challenges.

The mechanisms underlying the association between COPD and hepatic steatosis are likely multifaceted. Shared risk factors such as obesity, insulin resistance, and systemic inflammation are pivotal in both conditions^32,33^. Moreover, inflammatory cytokines like tumor necrosis factor-alpha and adipokines like leptin, which are often elevated in NAFLD, have been implicated in the progression of liver disease and also exert biological effects in other tissues and conditions, including COPD^34–36^. Our findings resonate with these mechanistic insights, further emphasizing the complex interplay between these conditions. Our study's observation of a CAP value inflection point at 272.39 dB/m, corresponding to the lowest COPD prevalence, may reflect the complex interplay between body composition and the progression of chronic diseases. The concept of the 'obesity paradox,' which posits that certain levels of increased body fat may confer protective effects in chronic diseases, appears relevant here. This paradox is underpinned by a better metabolic reserve in overweight individuals, which might offer a survival advantage in the face of chronic illnesses^37,38^. The significance of this paradox becomes particularly pronounced in elderly populations, where maintaining an 'optimal' BMI—rather than a lower BMI—is associated with reduced disease prevalence and mortality rates. This protective role of moderate adiposity, which CAP indirectly assesses through the quantification of hepatic fat content, may preserve muscle mass and nutritional status, both of which are crucial in managing COPD and other chronic conditions^39^. Therefore, the CAP value's inflection point identified in our study does not merely serve as a numerical marker but rather as a reflection of the intricate balance between adiposity levels and their impacts on chronic diseases like COPD. It underscores the importance of considering CAP within the broader context of the obesity paradox and its implications for patient care. This observation, linking CAP with the broader phenomena of the obesity paradox, invites a deeper exploration into the roles of body composition and metabolic health in the management and prognosis of COPD, thereby highlighting the potential utility of CAP as a tool for nuanced patient assessment beyond liver health alone.

This study has several strengths, including the large sample size, the use of VCTE for liver disease assessment, and the application of a nationally representative dataset. However, limitations must be noted. The cross-sectional design precludes causal inference, and the absence of a control group limits direct comparison with the general population. Furthermore, while VCTE is a robust tool, it does not replace the gold standard of liver biopsy for NAFLD diagnosis.

## Conclusion

In conclusion, our study highlights a significant and complex association between hepatic steatosis and COPD, underscoring the need to consider liver health in COPD management. Future research should focus on elucidating the mechanisms driving this association and on developing effective prevention and treatment strategies (“Supplementary information”).

## Supplementary Information

Supplementary Information.

## Data Availability

The survey data are publicly available on the internet for data users and researchers throughout the world (www.cdc.gov/nchs/nhanes/).

## Abbreviations

MASLD: Metabolic dysfunction-associated steatotic liver disease
VCTE: Vibration-Controlled Transient Elastography
COPD: Chronic obstructive pulmonary disease
NHANES: National Health and Nutrition Examination Survey
NCHS: National Center for Health Statistics
GOLD: Global Initiative for Chronic Obstructive Lung Disease
MAFLD: Metabolic associated fatty liver disease
NAFLD: Non-alcoholic fatty liver disease
LSM: Liver stiffness measurement
DEXA: Dual-energy X-ray
CAP: Controlled attenuation parameter
BMI: Body mass index
FEV1: 1 Second
FVC: Forced vital capacity
RCS: Restricted cubic spline
SD: Standard deviations
