# Author Correction: Association of hepatic steatosis and liver fibrosis with chronic obstructive pulmonary disease among adults

**DOI:** 10.1038/s41598-025-03794-y

**Published:** 2025-06-11

**Authors:** Dayang Zheng, Xiang Liu, Wei Zeng, Wangyan Zhou, Chunxiang Zhou

**Affiliations:** 1 Department of Thoracic Surgery, East Hospital, The Second Affiliated Hospital, Hengyang Medical School, University of South China, No. 30 Jiefang Road, Shigu District, Hengyang, 421009 Hunan Province China; 2 Department of Medical Record, The First Affiliated Hospital, Hengyang Medical School, University of South China, Hengyang, 421001 China

Correction to: *Scientific Reports* 10.1038/s41598-024-61696-x Published online 11 May 2024.

The original version of this Article contained an error in the Method section. Due to an error while drafting the Article, a wrong method for the classification of COPD was described.

In the Methods section, under the subheading ‘COPD’, the paragraph,

“If any of the following criteria were met, the diagnosis of COPD was established^27^: (1) participants exhibited a ratio of forced expiratory volume in 1 s (FEV1) to forced vital capacity (FVC) less than 0.70 after the administration of β2-adrenergic bronchodilator medication; (2) participants received a diagnosis of emphysema from a physician or other healthcare professional; and (3) participants aged 40 or above with a history of smoking and chronic bronchitis, who were using medications such as selective phosphodiesterase-4 inhibitors, mast cell stabilizers, leukotriene modifiers, and inhaled corticosteroids. In the assessment of COPD severity, we adopted the Global Initiative for Chronic Obstructive Lung Disease (GOLD) classification^28^. This framework categorizes COPD into four stages based on the severity of airflow limitation, which is determined by the post-bronchodilator FEV1/FVC ratio:

- GOLD 1 (Mild): FEV1 ≥ 80% predicted
- GOLD 2 (Moderate): 50% ≤ FEV1 < 80% predicted
- GOLD 3 (Severe): 30% ≤ FEV1 < 50% predicted
- GOLD 4 (Very Severe): FEV1 < 30% predicted”

now reads:

“Participants were classified as having COPD if they reported ever being told by a healthcare professional that they had COPD, chronic bronchitis, or emphysema. This classification method has been widely applied in previous NHANES-based research for identifying individuals with COPD.”

As a result, References 27 and 28 have been removed from the Reference list and subsequent references have been renumbered.

The original Article has been corrected.

