# Supplementary material for: Association of hepatic steatosis and liver fibrosis with chronic obstructive pulmonary disease among adults
Source: Sci Rep. 2024 May 11;14:10822. doi: 10.1038/s41598-024-61696-x (PMC11088642; doi:10.1038/s41598-024-61696-x)
Supplement: Supplementary file 1 — Supplementary Information. [file 41598_2024_61696_MOESM1_ESM.docx]

**Table S1:** The associations between CAP and GOLD-graded COPD.

| **GOLD Stage** | **Odds Ratio (OR) for CAP per 10 dB/m Increase** | **95% CI** |
| --- | --- | --- |
| Non-COPD | Ref. | Ref. |
| GOLD 1 (Mild) | 1.06 | (1.02, 1.10) |
| GOLD 2 (Moderate) | 1.05 | (1.00, 1.10) |
| GOLD 3 (Severe) | 1.03 | (0.98, 1.08) |
| GOLD 4 (Very Severe) | 1.02 | (0.95, 1.09) |

Age, gender, race, BMI, smoking status, ALT, ALP, AST, diabetes, PIR, high blood pressure, asthma, vigorous activities, and alcohol drinking were adjusted.
